# Gastric adenocarcinoma of fundic gland type arising from heterotopic gastric glands during a 19-year follow-up period

**DOI:** 10.1007/s12328-019-00989-5

**Published:** 2019-06-04

**Authors:** Takeshi Uozumi, Hideyuki Seki, Emi Matsuzono, Susumu Sogabe, Nozomu Sugai, Jun Fujita, Junichi Suzuki, Mayuko Akimoto, Mitsuru Yanai, Akira Suzuki

**Affiliations:** 1 Departments of Gastroenterology, KKR Sapporo Medical Center, 6-3-40, Hiragishi 1jou, Toyohira-ku, Sapporo, Hokkaido 062-0931 Japan; 2 Department of Pathology, KKR Sapporo Medical Center, Sapporo, Japan

**Keywords:** Gastric adenocarcinoma of fundic gland type, Heterotopic gastric gland, Gastric adenocarcinoma of fundic mucosa type, Endoscopic submucosal dissection, Long-term

## Abstract

A 73-year-old man with prior history of duodenal ulcer has been undergoing periodic upper gastrointestinal endoscopy since 1999. In 2017, a 25-mm submucosal tumor-like protrusion was detected in the lesser curvature of the upper stomach; histological examination of the lesion revealed gastric adenocarcinoma of fundic gland type. En bloc resection was achieved using endoscopic submucosal dissection. The patient was histopathologically diagnosed with gastric adenocarcinoma of fundic gland type arising from heterotopic gastric glands. Immunohistochemical staining was positive for MUC5AC, MUC6, pepsinogen I, and proton pump but negative for MUC2 and CD10. Moreover, the patient’s Ki-67 labeling index score was extremely low. The presence of MUC5AC indicated that the tumor differentiated to the foveolar epithelium and fundic glands. Gastric adenocarcinoma of fundic gland type that differentiates to several directions has a higher malignant potential than the disease that differentiates to chief cells. A retrospective review of the patient’s previous endoscopic examination revealed that the submucosal tumor-like protrusion existed since 2000; tumor size increased from 8 mm in 2000 to 25 mm in 2017. The present case is rare in that the carcinoma arose from heterotopic gastric glands. Moreover, the 19-year follow-up revealed that the tumor differentiated to the foveolar epithelium, considered as having high-grade malignancy.

## Introduction

Gastric adenocarcinoma of fundic gland type (GAFG) was proposed as a new histologic type of gastric cancer by Ueyama et al. in 2010 [1]. It was first reported that this condition manifests low-degree atypia and low-grade malignancy. However, as several case reports of GAFG were published, some showed high-degree atypia and high-grade malignancy [1, 2]. Heterotopic gastric gland (HGG) is a paracancerous lesion, and two case reports have shown GAFG co-existing with HGG [3, 4].

Herein, we report a case of a patient with GAFG that arose from HGG and differentiated to the foveolar epithelium.

## Case report

**Fig. 1:**
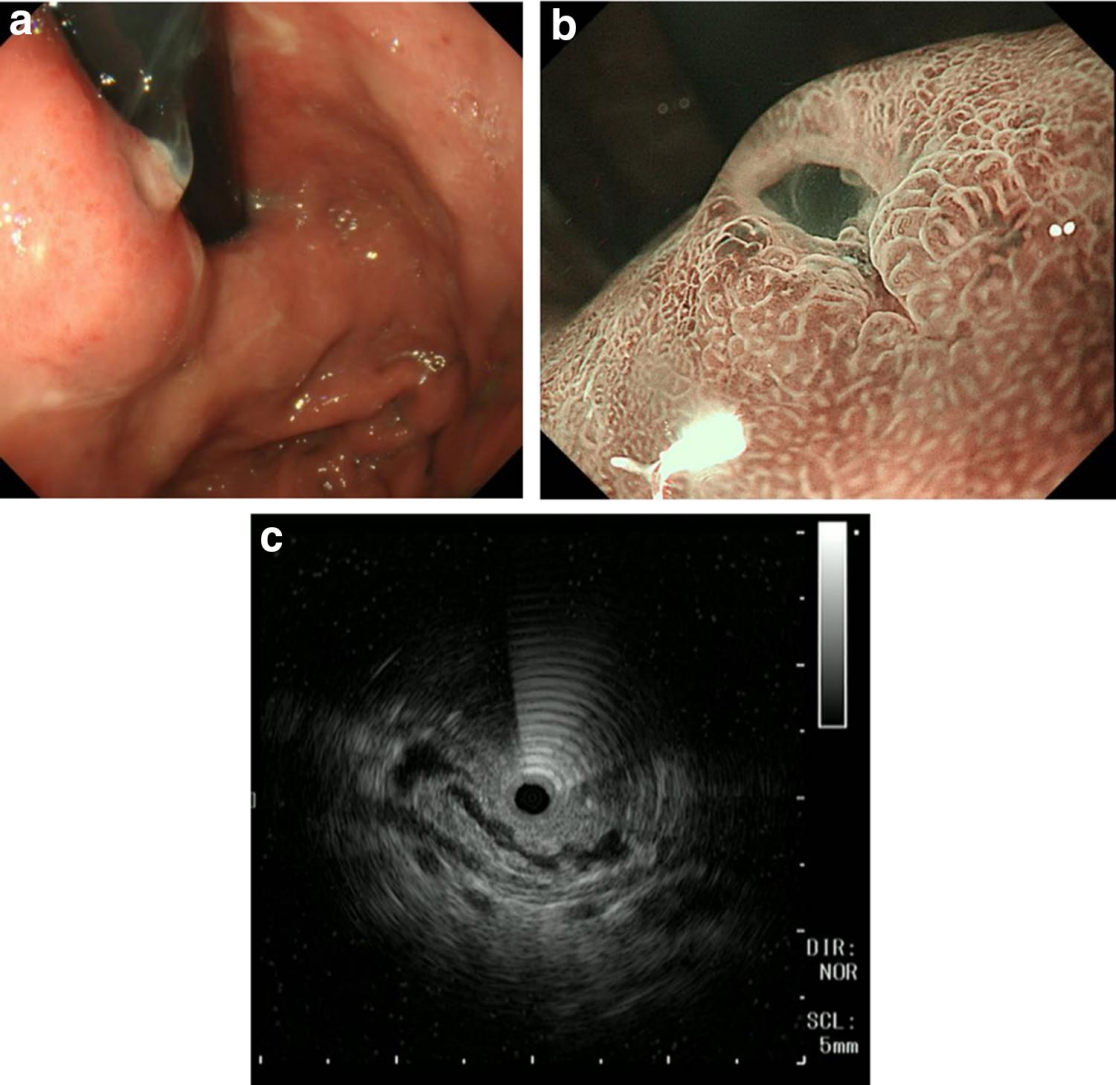
**a** A 25-mm submucosal tumor-like protrusion shows a slit-like opening at the top. **b** Dilation of the intervening portion between the crypts is observed on magnifying endoscopy with a narrow-band imaging. Regular surface and microvascular patterns without a demarcation line are noted. **c** With an endoscopic ultrasonography, low echoic lesion is observed in the third layer; however, the fourth layer remains intact

**Fig. 2:**
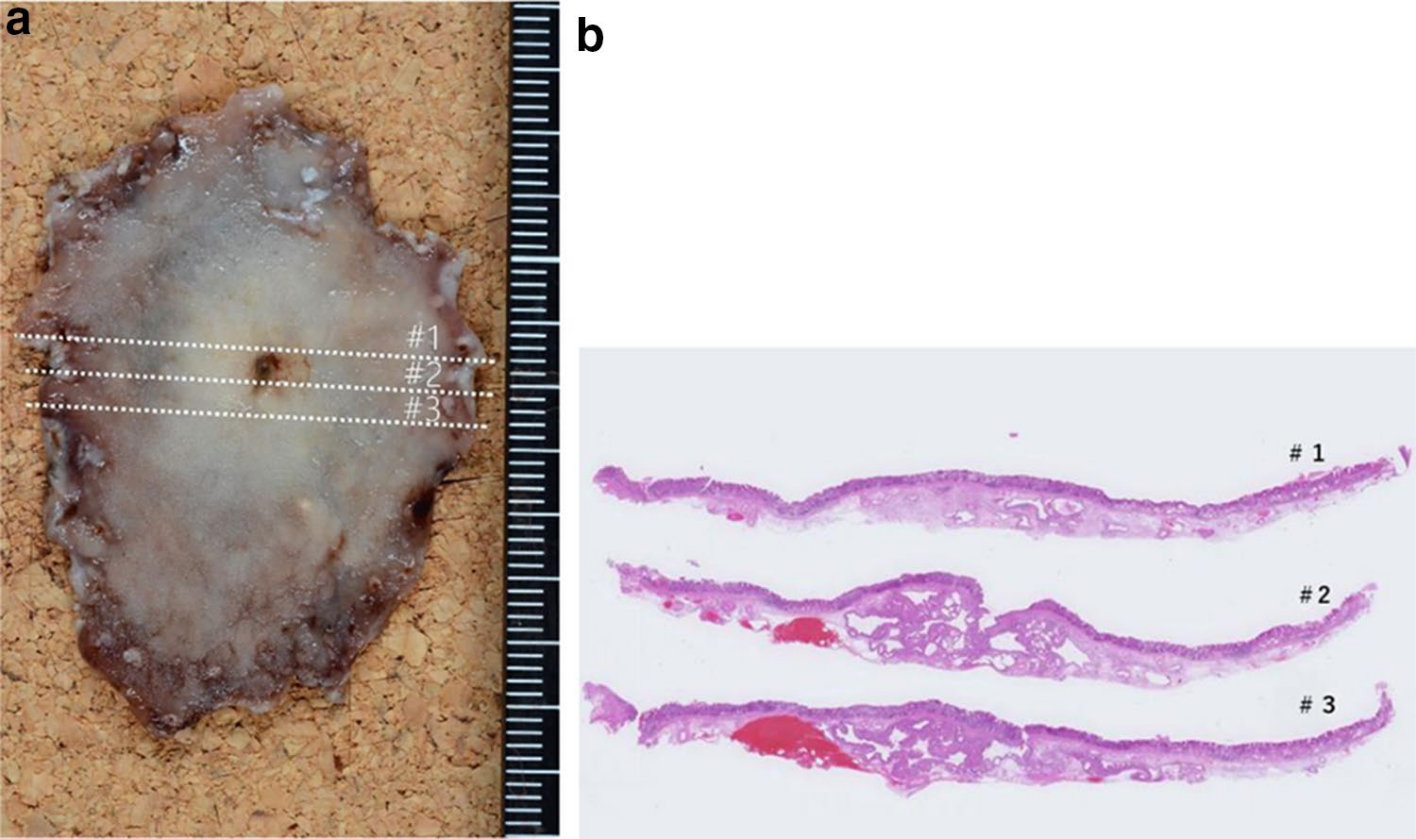
Histopathological features of the specimen obtained via endoscopic submucosal dissection

A 73-year-old man with prior history of duodenal ulcer has been undergoing periodic upper gastrointestinal endoscopy (UGI) since 1999. In 2017, a 25-mm submucosal tumor (SMT)-like protrusion with a slit-like opening at the top was detected in the lesser curvature of the patient’s upper stomach. Moreover, mucus oozed out from the slit (Fig. 1a). In the stomach, grade 0–1 atrophic gastritis was observed according to the Kimura–Takemoto classification [5], and the patient tested positive for anti-*Helicobacter pylori* immunoglobulin G antibody. Magnifying endoscopy with narrow-band imaging (M-NBI) revealed regular microsurface and microvascular patterns without a demarcation line according to the vessel plus surface (VS) classification system (Fig. 1b) [6]. GAFG was suspected following the histological examination of the biopsy specimen, which showed mimicking chief cells with a low degree of atypia in the submucosal layer. An endoscopic ultrasonography (EUS) performed revealed a hypoechoic mass in the third layer. However, the fourth layer was preserved (Fig. 1c). Therefore, we considered the deepest part of the tumor as the submucosal layer. Computed tomography did not reveal any metastasis. Although the tumor was now suspected as GAFG, a definite diagnosis was not achieved via histological examination. Informed consent was obtained from the patient, and we planned to perform endoscopic submucosal dissection (ESD) for total biopsy and treatment. En bloc resection achieved using ESD (Fig. 2).

**Fig. 3:**
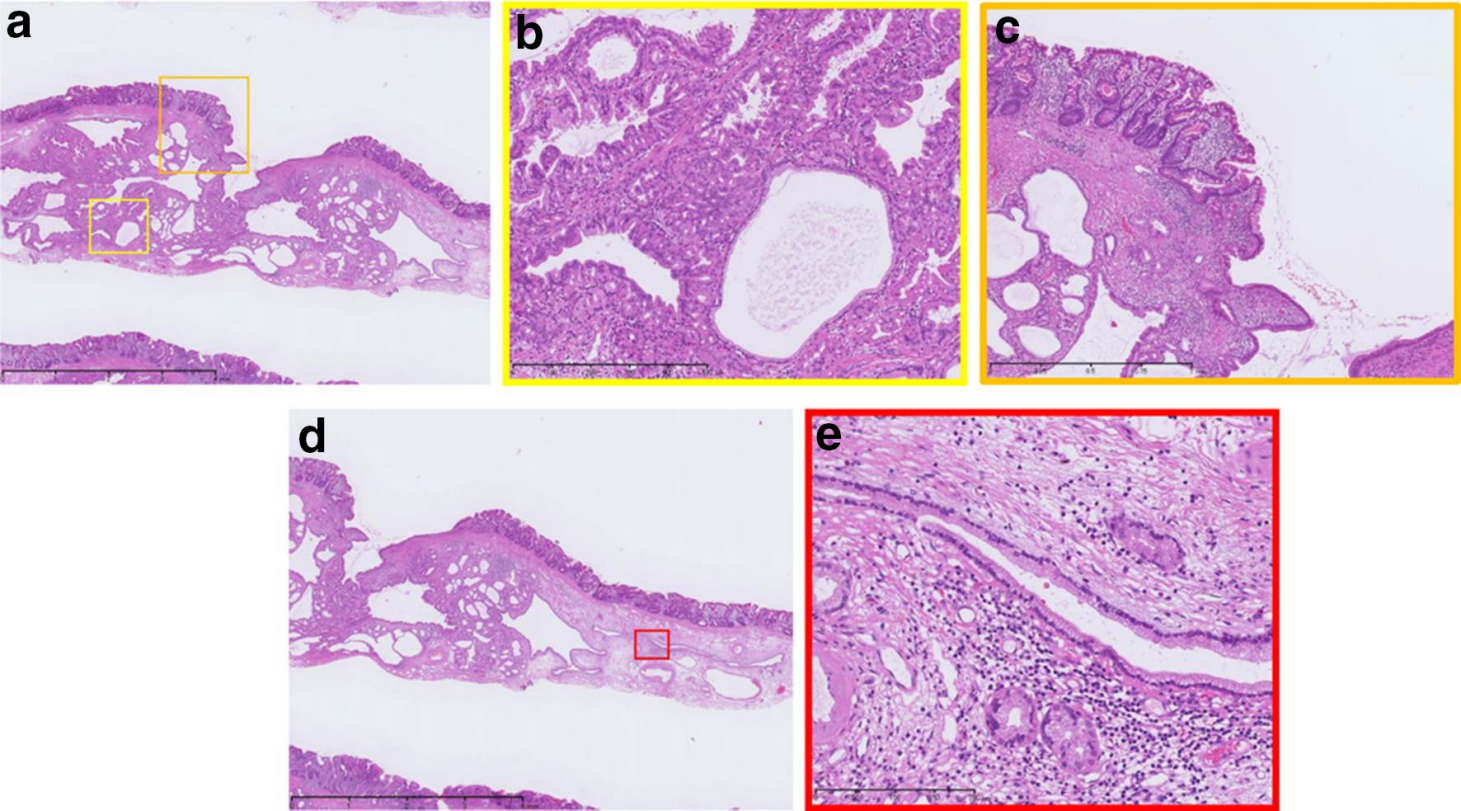
**a** Low-magnification image of specimen #2 shows the spread of cystic ducts and adenocarcinoma. **b** Magnification image of the yellow square. Disorder of nuclear polarity and atypia of the structure are observed. **c** Magnification image of the orange square. The surface of the tumor is covered with intestinal metaplasia, and no malignancy is observed in the mucosa. **d** Low-magnification image of specimen #2. The margin of the tumor is surrounded by cystic ducts. **e** Magnification image of the red square. The cystic ducts have a normal structure, and atypia is not observed

Histopathological examination of the resected specimen revealed the presence of intestinal metaplasia on the surface of the tumor; no neoplasia was observed in the mucosa (Fig. 3a, c). In the submucosal layer, irregularly shaped ducts including cribriform glands were detected that were formed by mimicking chief cells with atypia (Fig. 3b). Moreover, cystic ducts were observed in the margin of the tumor in the submucosal layer. However, the glands and cells had no atypia and proliferation (Fig. 3d, e).

**Fig. 4:**
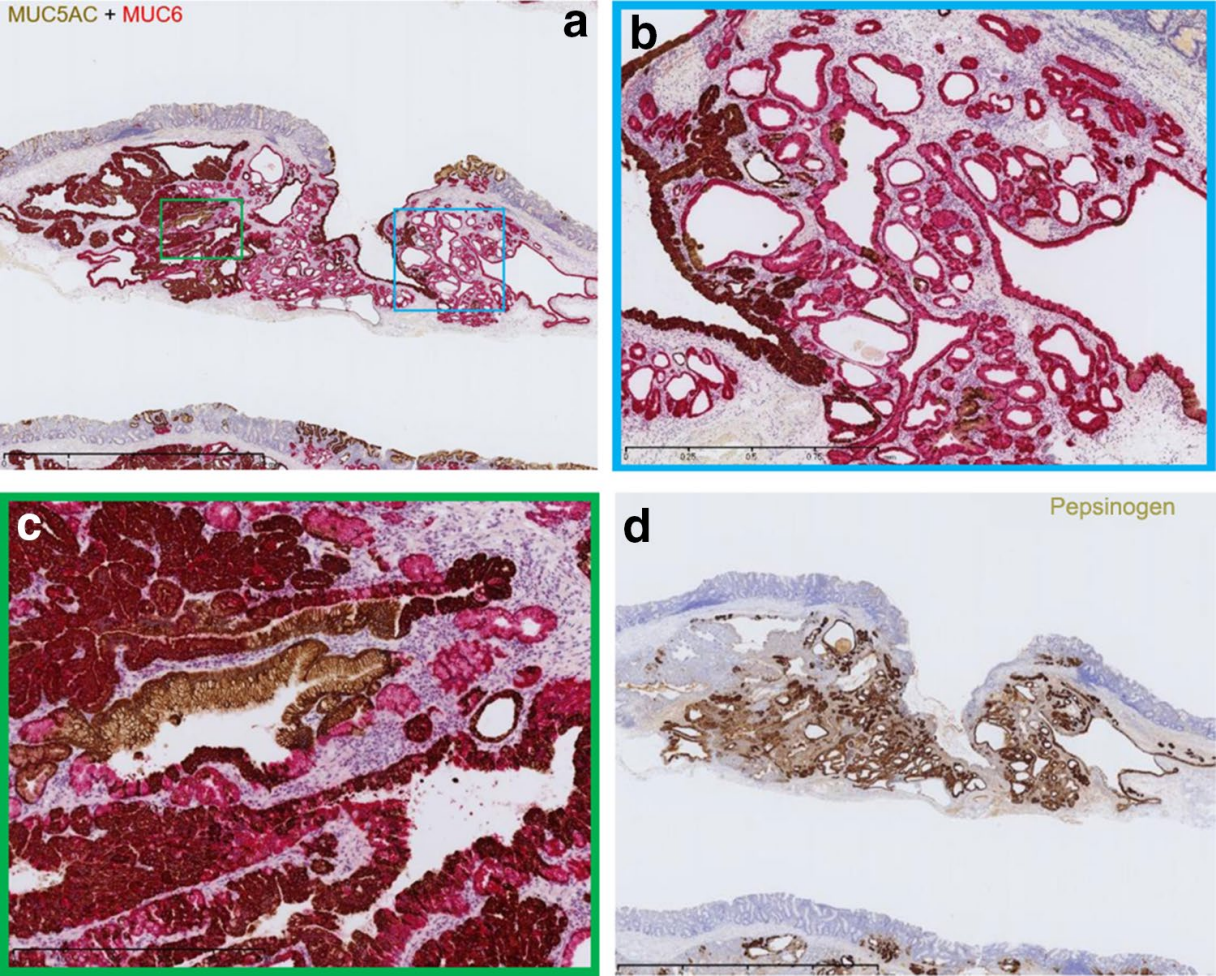
Immunohistochemical staining. **a** Double-stained image with MUC5AC (ochre) and MUC6 (purple). **b** Magnification image of the blue square. HGG is positive for MUC6 and negative for MUC5AC. **c** Magnification image of the green square. The area that is atypia in structure is positive for MUC5AC and MUC6. **d** Pepsinogen I staining. HGG is strongly positive for pepsinogen I

Immunohistochemical examination performed using antibodies to pepsinogen I, MUC5AC, MUC6, proton pump, CD10, MUC2, and Ki-67 confirmed that the irregularly shaped glands differentiated to the fundic gland and foveolar epithelium (Fig. 4). The Ki-67 labeling index score was extremely low. The glands in the margin of the tumor with low-degree atypia were positive for MUC6 and pepsinogen I but negative for MUC5AC.

The final post-ESD diagnosis was GAFG, and it was considered to primarily exist in the epithelium of the HGG in the submucosal layer. There was no invasion to submucosal stroma. According to the Japanese classification of gastric carcinoma, the tumor was classified as follows: gastric carcinoma of fundic gland type: U, LessPost, Type 0–IIa, 17 mm × 14 mm, pT1a(M), N0, M0, INFb, pUL0, ly0 (D2-40), v0 (Elastica-HE), pHM0, and pVM1 [7]. Neoplastic ducts were observed on the vertical margin of the ESD specimen. Therefore, the patient was informed regarding the risk of recurrence without additional resection. However, he decided not to undergo such procedure. At 3 months after ESD, no local recurrence was observed.

**Fig. 5:**
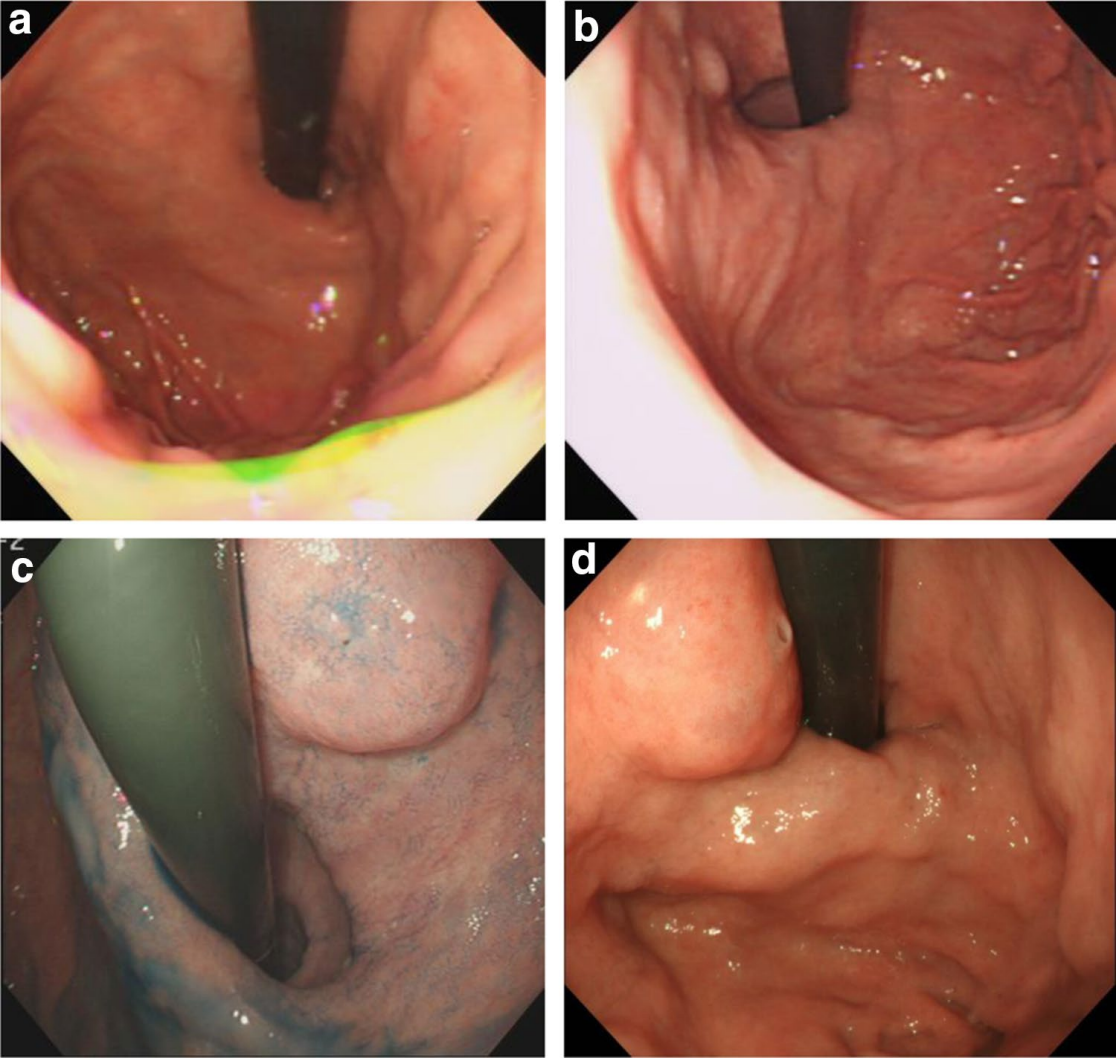
Previous upper gastrointestinal endoscopy examination results. **a** The tumor is absent in 1999. **b** An 8-mm elevated lesion is observed in the lesser curvature of the upper stomach in 2000. **c** A 15-mm elevated lesion is observed in the same place. No epithelial changes are noted in 2007. **d** A 20-mm elevated lesion is noted. The slit-like opening is observed at the top of the tumor, and mucus oozes from the opening in 2015

In the present case, UGI has been performed since 1999. A retrospective review of the past endoscopic examination results of this patient revealed no presence of a tumor until 1999 (Fig. 5a). However, an 8-mm SMT-like protrusion was detected in the lesser curvature of the upper stomach in 2000 (Fig. 5b). The tumor was 15 mm, and the elevation became clear in 2007 (Fig. 5c); however, the slit-like opening was absent during this examination. In 2015, the size of the tumor had increased compared with that in 2007 (Fig. 5d), and it was 20 mm in size. During this examination, the slit-like opening was observed at the top of the tumor. Mucus oozed from the slit.

## Discussion

GAFG is a well-differentiated tubular adenocarcinoma comprising various mildly atypical columnar cells that mimic the fundic glands. MUC6 and pepsinogen I are strongly expressed, and typical GAFG is negative for CD10 and MUC2 [1, 8].

Previously, GAFG was considered to have a low-grade malignancy [1]. In one case, the 12-year natural history of GAFG was observed, and no morphological changes were observed [9]. The case report supports the notion that GAFG is an adenocarcinoma of low-grade malignancy. In contrast, in some cases, high-degree atypia and high-grade malignancy have been reported [2], and it was speculated that GAFG that differentiated to several directions had a higher malignant potential.

In Japan, Tanabe et al. [10] have reported about gastric adenocarcinoma of fundic gland mucosa type (GAFGM) that showed atypical cells that differentiated to the fundic gland and foveolar epithelium. Based on immunohistochemical examination, individuals with GAFGM tested positive for MUC5AC. Six cases of GAFGM were reported, and all showed infiltration into the submucosal layer. The prognosis of GAFGM may be worse than that of the typical GAFG [10].

The present patient tested positive for MUC6 and pepsinogen I; moreover, mimicking of the chief cell was observed. Therefore, the tumor was diagnosed as GAFG. The tumor was positive for MUC5AC, and it may be diagnosed as GAFGM according to the above-mentioned report.

The etiology of HGG is considered the aberration of the epithelium into the submucosa as a result of repeated erosion and regeneration of the mucosa. HGG and gastric adenocarcinoma develop as a result of repeated erosion and regeneration of the mucosa, and this fact suggests that HGG is a paracancerous lesion [11–13]. Only two case reports have shown GAFG to be associated with HGG [3, 4]. In one of these reports, the patient had tested positive for MUC5AC and was diagnosed with GAFGM [4].

We concluded that the GAFG arose from the HGG based on two points. First, the GAFG existed only in the submucosal layer and was surrounded by HGG without neoplastic changes. Second, there were no neoplastic changes in the mucosal layer over the tumor. Although the cancer existed under the muscularis mucosa, it was diagnosed as mucosal cancer based on its depth, as it was limited to the epithelium of the HGG and did not show any invasion of the submucosal stroma. The size of the tumor increased from 1999 to 2017. In 2015, a slit-like opening was observed on the upper side of the tumor for the first time. We suggest that the change was caused by conversion to malignant tumor. GAFG arising from HGG may have increased the tumor size in the submucosal layer, and the mucus accumulated in the HGG oozed from the upper side of the tumor.

Endoscopic finding of GAFG with M-NBI does not usually meet the criteria for the diagnosis of carcinoma using the VS classification system. The following features have typically been observed using M-NBI: (1) an indistinct line of demarcation between the lesion and surrounding mucosa, (2) the dilatation of the crypt opening, (3) the dilatation of the intervening portion between the crypts, and (4) microvessels without distinct irregularities [14]. These features appear due to the location of the tumor origin. However, GAFG that is positive for MUC5AC differentiates to the foveolar epithelium, and thus, shows epithelial changes and meets the criteria for the diagnosis of carcinoma [15]. In the present patient, the tumor existed primarily in the submucosal layer. Therefore, no changes were observed in the epithelium, and the tumor did not meet the criteria.

GAFG that differentiates to several directions is considered to have higher malignancy than the chief cell dominant type; however, the prognosis is unknown [1, 8, 10]. In the present patient, the tumor differentiated to the foveolar epithelium. However, the tumor indicated extremely low Ki-67 labeling index and it did not show invasion to the submucosal stroma in the long term. We believe that this case report is valuable as no other case report has observed the long-term natural course of GAFG with high-grade malignancy yet.

The limitation of this case report is that biopsy was not previously performed; therefore, we could not identify when the GAFG arose from HGG.
